# Genetic profile and biological implication of PIN2/TRF1-interacting telomerase inhibitor 1 (PinX1) in human cancers: an analysis using The Cancer Genome Atlas

**DOI:** 10.18632/oncotarget.18589

**Published:** 2017-06-21

**Authors:** Wei-Juan Huang, Mei Li, Xiao-Han Jin, Xiao-Jia Huang, Wei Zhao, Xiao-Peng Tian

**Affiliations:** ^1^ Department of Histology and Embryology, Zhongshan School of Medicine, Sun Yat-Sen University, Guangzhou, China; ^2^ Department of Pharmacology, Jinan University, Guangzhou, China; ^3^ Department of Pathology, Cancer Center, Sun Yat-Sen University, Guangzhou, China; ^4^ The State Key Laboratory of Oncology in South China, Sun Yat-Sen University Cancer Center, Collaborative Innovation Center for Cancer Medicine, Guangzhou, China

**Keywords:** PinX1, cancer, expression pattern, function, cBioportal

## Abstract

Pin2/TRF1-interacting telomere inhibitor 1 (PinX1) was originally identified as a telomerase inhibitor, involved in maintaining telomerase activity, telomere length, and chromosomal stability. However, research has shown that PinX1 can have opposing molecular status in its expression patterns in several other tumor types. We thus investigated the genetic profile and biological implication of PinX1 in several human cancers using the cBioportal database. Our results showed that *PinX1* deletion accounted for the most alterations, with the frequency of its deletion regularly occurring in pathological types of carcinosarcoma and adenocarcinoma. We found few instances of *PinX1* gene mutations and 3D structural analysis demonstrated that these mutation sites were always located within telomerase inhibitor domains. Furthermore, our analysis of several human cancers from the cBioportal database revealed more frequent *PinX1* homozygous depletion and *PinX1* heterozygous deficiency, but both more infrequent *PinX1* gain and rare instances of *PinX1* amplification. The status of *PinX1* genetic alterations was correlated with prognosis and may be tumor-type specific. As such, its biological function in tumorigenesis and later prognosis is complicated and may involve co-worked with NEIL2, R3HCC1, POLR3D, GTF2E2, and INTS10. In addition, we observed that PinX1 interacts with TERT, DKC1, PTGES3, and HSP90AA1. PinX1 mRNA expression was decreased in most selected cancer tissues, which could promote tumor growth and enhance tumorigenicity. Collectively, our data reveal PinX1 expression patterns and potential mechanisms in various human cancers. Further work will be needed to comprehensively examine its role in tumor genesis and progression.

## INTRODUCTION

Telomeres cap the ends of chromosomes and are critical for maintaining genomic integrity. Many studies have shown that telomere shortening or dysfunction is often acquired during the process of tumorigenesis [1]. Telomerase is the natural enzyme complex that elongates telomeres, doing so through the addition of telomere repeats at chromosomal ends. It plays a crucial role in maintaining telomere length, which has been implicated in human cancer tumorigenesis through the maintenance of genomic stability and avoidance of senescence [2, 3].

Telomerase activity is responsible for the hallmark phenotype of human tumors—immortalization [4]. It has been detected in approximately 80% of human tumors and identified as a key step in human cellular tumorigenesis [5]. The interplay between telomerase and the telomere is regulated by the shelterin complex, which binds to and protects telomeres at chromosomal ends [6]. The shelterin complex is composed of six telomere-specific proteins, including: Telomere Repeat Factor 1 (TRF1), Telomere Repeat Factor 2 (TRF2), TRF-1 and TRF-2 Interacting Nuclear Factor 2 (TIN2), Transcriptional Repressor/Activator Protein 1 (RAP1), POT1-Organizing Protein (TPP1), and protection of telomere 1 (POT1) [7]. Communication with the shelterin complex is an essential step for telomerase to carry out its functions in telomere maintenance.

PIN2/TRF1-interacting telomerase inhibitor 1 (PinX1) was first identified as a Pin2/TRF1 binding protein [8]. This conserved nuclear protein is a potent telomerase inhibitor and a putative tumor suppressor. Its telomerase inhibitory domain (TID) is located at the region of 254aa-328aa. Functionally, it binds to the telomerase catalytic subunit hTERT and potently inhibits its activity [9]. Many studies have shown that PinX1 can regulate telomere maintenance in cancer cells, ultimately inhibiting telomere elongation [10]. Additional work has shown that depletion of endogenous PinX1 or its TID domain elongates telomeres, induces immortalization, and increases tumorigenicity [11]. Furthermore, PinX1 knockdown leads to chromosomal instability, as its loss can cause delayed mitotic entry during mitosis, thereby abrogating faithful chromosome segregation [12].

However, the molecular mechanism(s) behind PinX1 remain unclear. Recently, our group discovered that that not only does PinX1 contribute to telomere maintenance, but it also affects cancer cell sensitivity to DNA damage caused by chemo-radiotherapy or chemotherapeutics [13]. In addition, the pattern of *PinX1* genetic expression is vastly different in various tissues and tumor types. For instance, Cai et al. demonstrated that loss of PinX1 was correlated to patients with poorer prognoses, suggesting that insufficient PinX1 may be a tumorigenic factor [14]. Other studies have demonstrated that PinX1 expression was upregulated in esophageal squamous cell carcinoma (ESCC) as well as cervical squamous cell carcinomas (CSCC) tissue, suggesting that abnormal PinX1 gene regulation and/or protein functions in tumorigenesis are complicated and are likely be tumor-type-specific [15]. Given this, we sought to investigate the biological and clinicopathological significance of PinX1 in various malignancies using the cBioportal database. This was done to better understand the potential role played by PinX1 in tumor development.

## RESULTS

### *PinX1* gene alteration in 105 studies using the online resource cBioportal web

**Figure 1:**
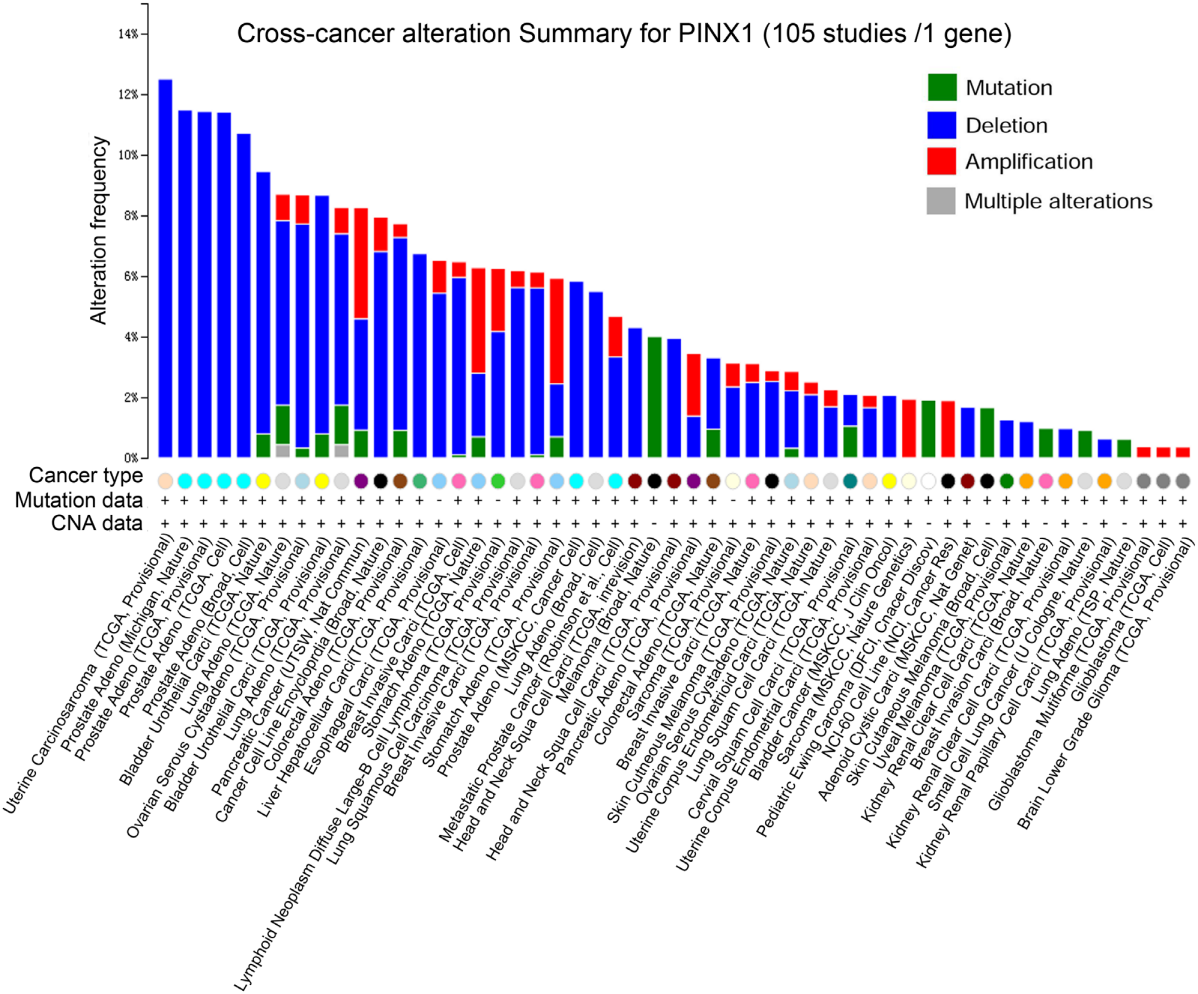
*PinX1* gene alteration in 105 studies selected from cBioportal Four alterations (Mutation, Deletion, Amplification, and Multiple alterations) were detected and visualized in 53 separate studies.

PinX1 gene alterations were detected in 105 separate studies using the online resource cBioportal Web. As shown in Figure 1, four alterations (Mutation, Deletion, Amplification, and Multiple Alterations) were detected and visualized in 53 studies. *PinX1* deletion accounted for the most alterations, with the highest ratio of 12.5% (Uterine Carcinosarcoma, TCGA, Provisional). Furthermore, the frequency of *PinX1* deletion often occurred in the two specific pathological types of carcinosarcoma and adenocarcinoma. This accounted for almost all pathology types with an alteration frequency of more than 6%. In addition, the frequency of *PinX1* gene deletion in adenocarcinoma was more than squamous carcinoma in some solid tumors, such as 6.1% (TCGA, Nature), 5.5% (Broad, Cell), and 5.7% (TCGA, Provisional) in lung adenocarcinoma versus 1.7% (TCGA, Provisional) in lung squamous cell carcinoma.

### *PinX1* genetic mutation levels in 105 studies using cBioportal web

**Figure 2:**
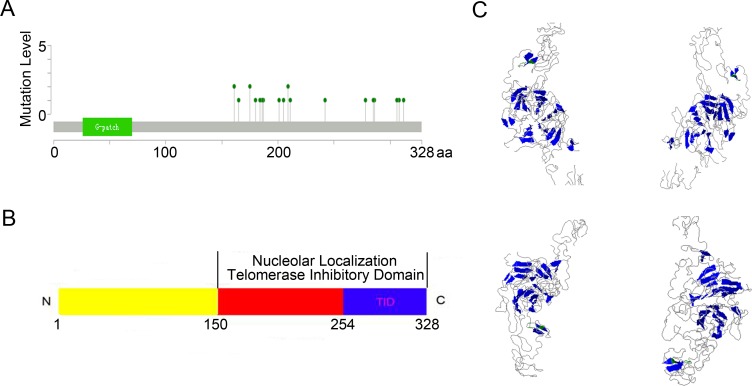
*PinX1* gene mutation level in 105 studies selected from cBioportal Web **(A)** Totally 33 mutations sites were detected and located between 150aa and 328aa. Only three sites (S161N/R, A175T, R209C/H) had reached mutation level 2 (the number of patients with the same mutation site). **(B** and **C)** Structural diagram and 3D structural analysis showed that these mutation sites were always located in the PinX1 function domain (nucleolar localization and telomerase inhibitor domains).

When compared with the high frequency of *PinX1* genetic deletions, there were few, frequent *PinX1* gene mutations the in 105 studies examined using cBioportal Web. As shown in Supplementary Data 1 and Figure 2A, a total of 33 mutation sites were detected and located between amino acids 150 and 328. Of these, only three sites (S161N/R, A175T, R209C/H) had reached mutation level 2 (the number of patients with the same mutation site). When combined with previous studies, 3D structural analysis showed that these mutation sites were always located in the PinX1 functional domain (nucleolar localization domain and telomerase inhibitor domain) (Figure 2B and 2C).

### *PinX1* genetic profile in six selected studies using cBioportal web

**Figure 3:**
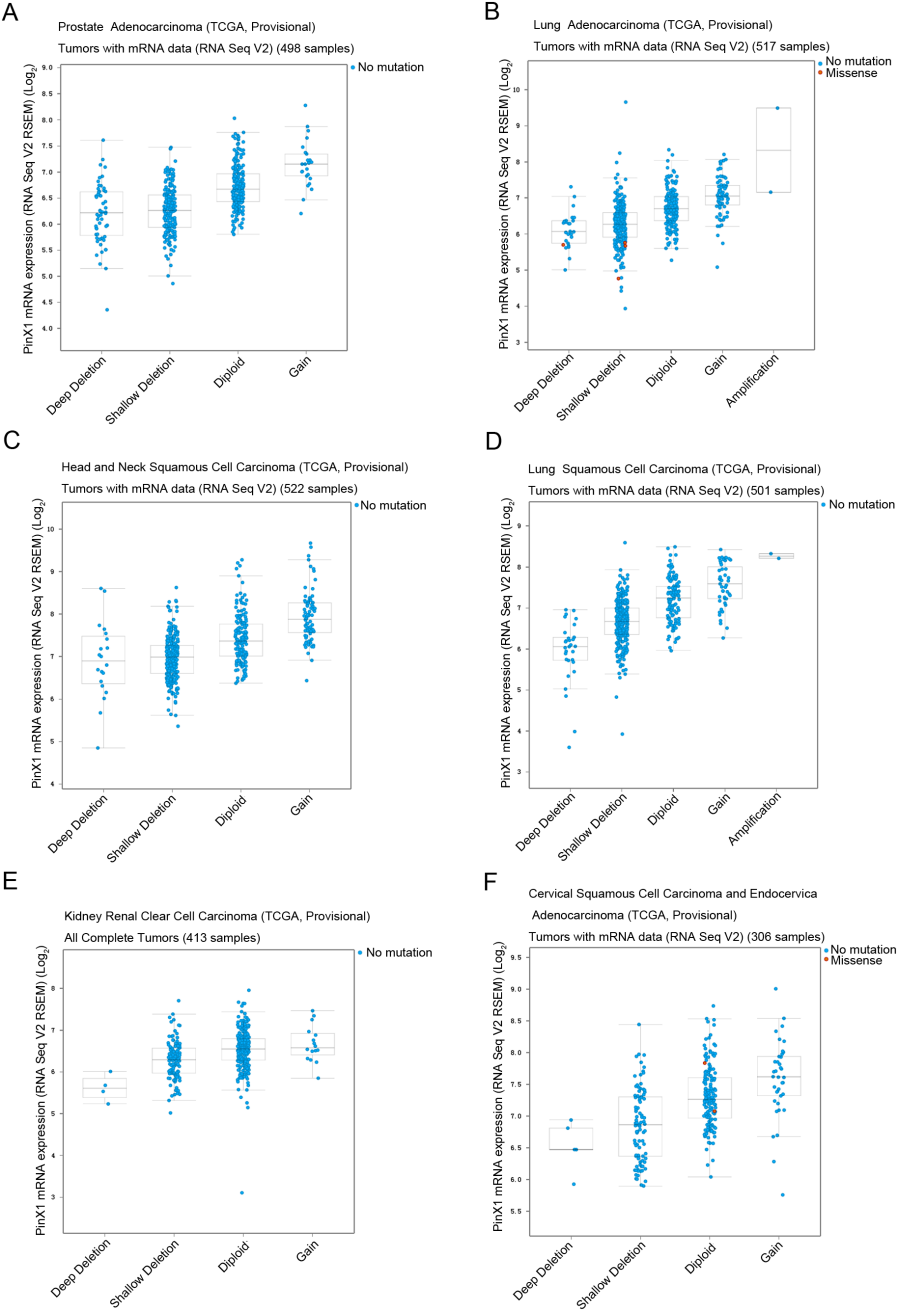
*PinX1* genetic profile in six types of cancer selected using cBioportal Relative expression levels as a function of relative *PinX1* gene copy number were plotted against six types of cancer. **(A)** prostate adenocarcinoma (TCGA, provisional); **(B)** lung adenocarcinoma (TCGA, provisional); **(C)** head and neck squamous cell carcinoma (TCGA, provisional); **(D)** lung squamous cell carcinoma (TCGA, provisional); **(E)** kidney renal clear cell carcinoma (TCGA, provisional); **(F)** cervical squamous cell carcinoma and endocervical adenocarcinoma (TCGA, provisional). Shallow deletion — heterozygously deleted; Diploid — two alleles present; Gain — low-level gene amplification event; Amplification — high-level gene amplification event.

The prostate adenocarcinoma (TCGA, Provisional) (Figure 3A), lung adenocarcinoma (TCGA, Provisional) (Figure 3B), head and neck squamous cell carcinoma (TCGA, Provisional) (Figure 3C), lung squamous cell carcinoma (TCGA, Provisional) (Figure 3D), kidney renal clear cell carcinoma (TCGA, Provisional) (Figure 3E), and cervical squamous cell carcinoma and endocervical adenocarcinoma (TCGA, Provisional) (Figure 3F) databases were selected to observe the relationship between *PinX1* gene copy number and mRNA levels. As shown in Figure 3A-3F, five types of copy numbers (deep deletion, shallow deletion, diploid, gain, amplification) were detected in six selected studies. More frequent *PinX1* homozygous depletion and *PinX1* heterozygous deficiency along with infrequent *PinX1* gain and rare PinX1 amplification ware obtained from these six databases. These results demonstrate that the frequency of *PinX1* homozygous depletion was often the main reason for the frequency of *PinX1* gene deletion in a wide variety of tumor tissues.

### *PinX1* gene alteration associated with overall survival and disease-free survival in eight selected studies using cBioportal web

**Figure 4:**
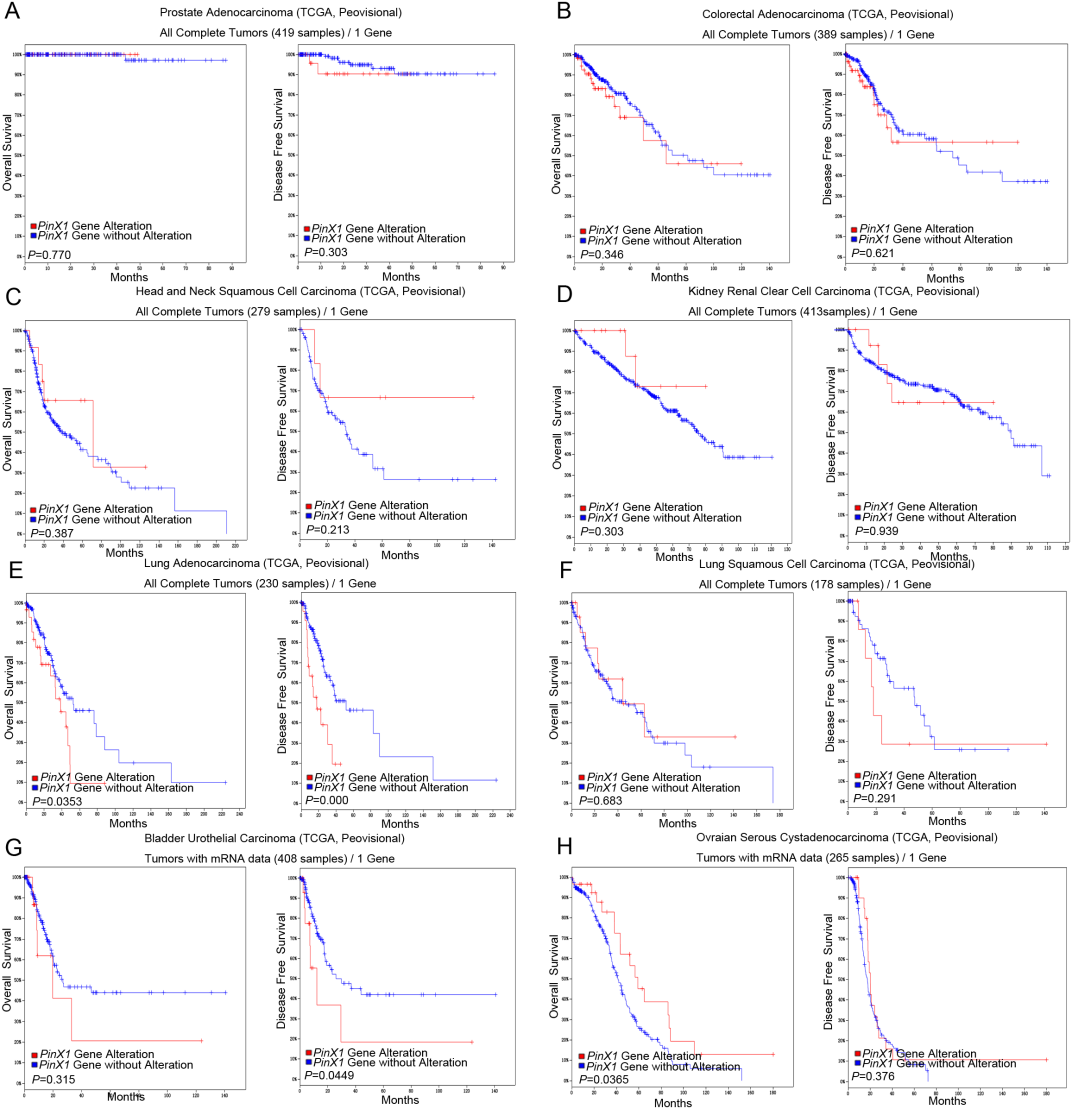
*PinX1* gene alteration associated with overall survival and disease-free survival in eight selective studies using cBioportal *PinX1* gene alterations associated with overall and disease-free survival rates in eight selected studies using cBioportal. **(A)** prostate adenocarcinoma (TCGA, provisional); **(B)** colorectal adenocarcinoma (TCGA, provisional); **(C)** head and neck squamous cell carcinoma (TCGA, provisional); **(D)** kidney renal clear cell carcinoma (TCGA, provisional); **(E)** lung adenocarcinoma (TCGA, provisional); **(F)** lung squamous cell carcinoma (TCGA, provisional); **(G)** bladder urothelial carcinoma (TCGA, provisional); **(H)** ovarian serous cystadenocarcinoma (TCGA, provisional).

The prostate adenocarcinoma (TCGA, Provisional), colorectal adenocarcinoma (TCGA, Provisional), head and neck squamous cell carcinoma (TCGA, Provisional), kidney renal clear cell carcinoma (TCGA, Provisional), lung adenocarcinoma (TCGA, Provisional), lung squamous cell carcinoma (TCGA, Provisional), bladder urothelial carcinoma (TCGA, Provisional) and ovarian serous cystadenocarcinoma (TCGA, Provisional) were randomly selected to observe the relationship between *PinX1* gene alteration and patient’s survival. As shown in Figure 4, there were no significant correlations between *PinX1* gene alteration and overall survival and/or disease-free survival in prostate adenocarcinoma (TCGA, Provisional) (*P*>0.05, Figure 4A), colorectal adenocarcinoma (TCGA, Provisional) (*P*>0.05, Figure 4B), head and neck squamous cell carcinoma (TCGA, Provisional) (*P*>0.05, Figure 4C), kidney renal clear cell carcinoma (TCGA, Provisional) (*P*>0.05, Figure 4D), and lung squamous cell carcinoma (TCGA, Provisional) (*P*>0.05, Figure 4F). There was also no significant correlation between *PinX1* gene alteration and overall survival in bladder urothelial carcinoma (TCGA, Provisional) (*P*>0.05, Figure 4G) and disease-free survival in ovarian serous cystadenocarcinoma (TCGA, Provisional) (*P*>0.05, Figure 4H). However, *PinX1* gene alteration was significantly correlated with poor overall survival and/or disease-free survival in lung adenocarcinoma (TCGA, Provisional) (*P*<0.05, Figure 4E), poor overall survival in bladder urothelial carcinoma (TCGA, Provisional) (*P*<0.05, Figure 4G), and poor disease-free survival ovarian serous cystadenocarcinoma (TCGA, Provisional) (*P*<0.05, Figure 4H). These results demonstrate that the relationship between *PinX1* status and prognosis may be tumor-type specific. Moreover, its biological function in tumorigenesis as well as prognosis is complicated and mainly co-regulated with other factors.

### *PinX1* gene correlations in seven selected studies using cBioportal web

Prostate adenocarcinoma (TCGA, Provisional), bladder urothelial carcinoma (TCGA, Provisional), lung adenocarcinoma (TCGA, Provisional), colorectal adenocarcinoma (TCGA, Provisional), hepatocellular carcinoma (TCGA, Provisional), head and neck squamous cell carcinoma (TCGA, Provisional), and lung squamous cell carcinoma (TCGA, Provisional) were selected to examine other gene correlations with *PinX1*. As shown in Table 1, ten of the most closely related (positively or negatively correlated) genes with *PinX1* are listed in their corresponding seven, selected studies. Our results showed that NEIL2, R3HCC1, POLR3D, GTF2E2, and INTS10 were the most common, positively correlated genes were *PinX1* within these studies. However, there were no common genes that were negatively correlated with *PinX1*.

**Table 1:** Ten of the most closely related (positively or negatively correlated) genes with *PinX1* are explored in cBioportal database

	Positive correlation with *PinX1* gene	Common Positive correlation with *PinX1* gene	Negative correlation with *PinX1* gene	Common Negative correlation with *PinX1* gene
Prostate Adenocarcinoma (TCGA, Provisional)	NEIL2, R3HCC1, MSRA, POLR3D, BIN3, UXT, RASSF1, C14ORF80,CD2BP2, SNF8, et al.	NEIL2, R3HCC1, POLR3D,GTF2E2, INTS10.	KAT6B, PDPK1, RBL2, TMEM184C, CHD6, CTNND1, MAPK8, RREB1, NR2C2, TGFBRAP1, et al.	None
Bladder Urothelial Carcinoma (TCGA, Provisional)	POLR3D, RAN, RPL26L1, NOP56, NTMT1, LYAR, NDUFAF2, GTF2E2, PTTG1, NPM3, et al.		KIAA1107, PIK3C2B, PLEKHA6, SCP2, ZNF611, CGN, ZNF91, ZNF254, ZNF816, ZNF217, et al.	
Lung Adenocarcinoma (TCGA, Provisional)	MCPH1, AGPAT5, NEIL2, INTS10, CNOT7, PSMA3, TTI2, R3HCC1, TIMM9, SEC61B, MAK16, et al.		ATXN1, AAK1.	
Colorectal Adenocarcinoma (TCGA, Provisional)	INTS10, GTF2E2, AGPAT5, CNOT7, INTS9, PBK, CCDC25, MCPH1, R3HCC1, BIN3, et al.		HECA, NR1D2, CTDSP2, PHC3, KLHL24, HDAC5, KBTBD2, CDK13, DVL3, RNF38, et al.	
Liver Hepatocellular Carcinoma (TCGA, Provisional)	NEIL2, R3HCC1, ERICH1, GTF2E2, LSM1, MSRA, DCTN6, TM2D2, TTI2, PPP2CB, et al.		ZNF609, DIP2B, CHD2, SP1.	
Lung Squamous Cell Carcinoma (TCGA, Provisional)	POLR3D, MCPH1, ERI1, CCDC25, ELP3, INTS10, PPP2R2A, AGPAT5, R3HCC1, DCTN6, BIN3, et al.		None	
Head and Neck Squamous Cell Carcinoma (TCGA, Provisional)	BIN3, ERI1, CCDC25, ELP3, CCAR2, SNRPA, PA2G4, AGPAT5, CNOT7, CDCA2, et al.		KIDINS220, JAK1, EFCAB14, IGF2R, MAPK3K2, LEPROT, SERINC1, ACBD3, BMPR2, CTDSP2, et al.	

### Integrated networks to evaluate PinX1 gene connectivity using cBioportal for cancer genomics

We next used the cBioportal for Cancer Genomics network to evaluate PinX1 gene connectivity. As shown in Supplementary Figure 1, PinX1 interacted with TERT, which is connected to both telomerase activity and telomerase stability as well as DKC1, which is connected to telomerase maintenance, DNA damage response, and cell adhesion. PTGES3 was also connected to PinX1, which is required for proper glucocorticoid and other steroid receptor functioning. Finally, HSP90AA1 was also shown to interact with PinX1, which mainly participates in stabilizing many proteins required for tumor growth.

### qRT-PCR analysis of PinX1 mRNA expression in nine human cancer types

**Figure 5:**
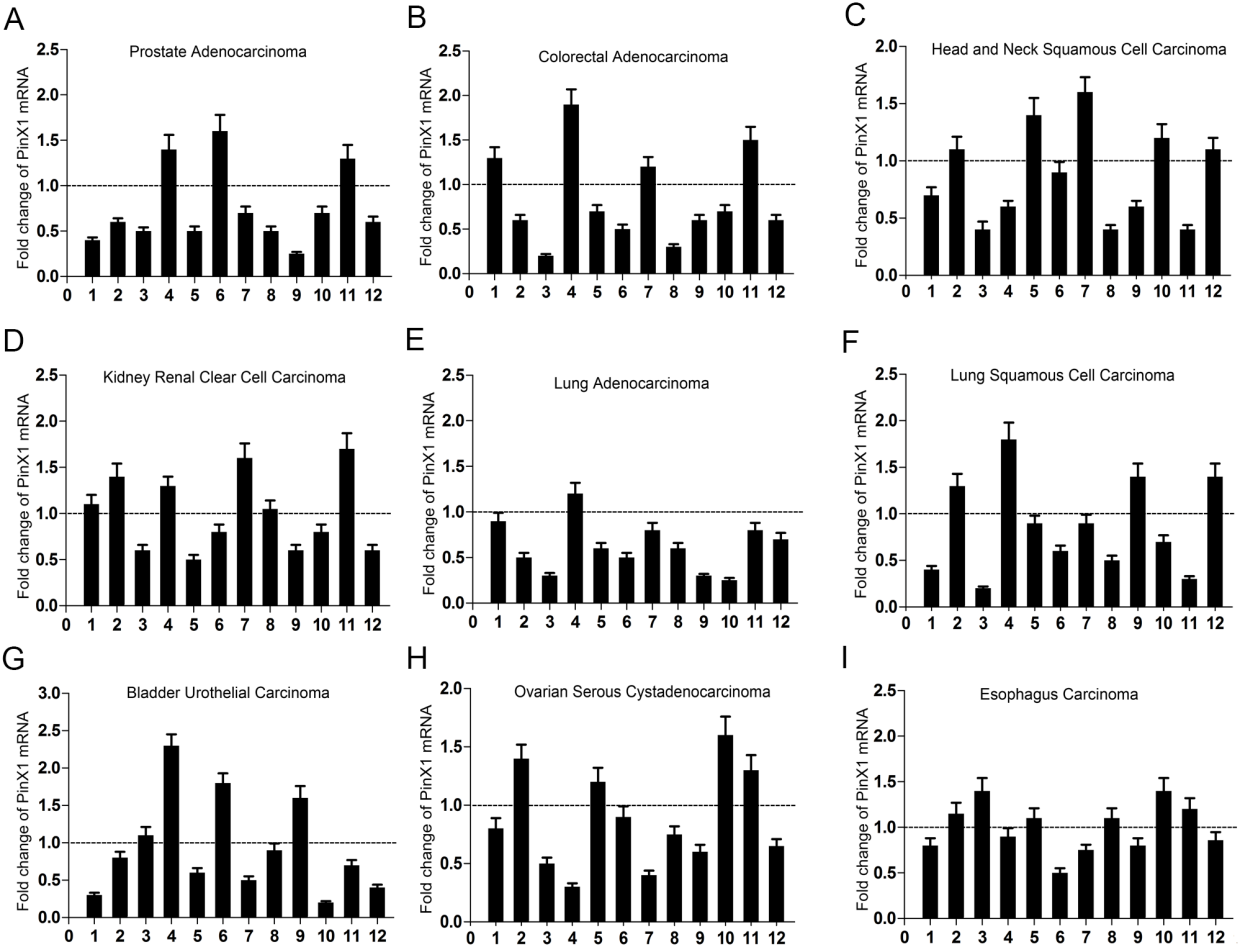
qRT-PCR used to analyze PinX1 mRNA expression status in nine types of human cancers Nine types of human cancer tissues were selected to analyze PinX1 mRNA expression using qRT-PCR. Low PinX1 mRNA expression was observed in 9 out of 12 (75%) prostate adenocarcinoma patient samples **(A)**, 8 out of 12 (66.7%) colorectal adenocarcinoma samples **(B)**, 7 out of 12 (58.3%) head and neck squamous cell carcinoma samples **(C)**, 7 out of 12 (58.3%) kidney renal clear cell carcinoma samples **(D)**, 11 out of 12 (91.7%) lung adenocarcinoma samples **(E)**, 8 out of 12 (66.7%) lung squamous cell carcinoma samples **(F)**, 8 out of 12 (66.7%) bladder urothelial carcinoma samples **(G)**, 8 out of 12 (66.7%) ovarian serous cystadenocarcinoma samples **(H)**, 6 out of 12 (50%) esophagus carcinoma tissues samples **(I)**. All cancerous tissue was compared to normal tissue samples.

To verify the PinX1 mRNA expression data found in our analysis of past studies in cBioportal for Cancer Genomic, we selected nine types of primary cancer patients’ tissues from our affiliated hospitals. From these selected tissues, we observed PinX1 mRNA expression in 9 out of 12 (75%) prostate adenocarcinoma patient samples (Figure 5A), 8 out of 12 (66.7%) colorectal adenocarcinoma samples (Figure 5B), 7 out of 12 (58.3%) head and neck squamous cell carcinoma samples (Figure 5C), 7 out of 12 (58.3%) kidney renal clear cell carcinoma samples (Figure 5D), 11 out of 12 (91.7%) lung adenocarcinoma samples (Figure 5E), 8 out of 12 (66.7%) lung squamous cell carcinoma samples (Figure 5F), 8 out of 12 (66.7%) bladder urothelial carcinoma samples (Figure 5G), 8 out of 12 (66.7%) ovarian serous cystadenocarcinoma samples (Figure 5H), and 6 out of 12 (50%) esophagus carcinoma tissues samples (Figure 5I). All samples were evaluated by qRT-PCR in comparison to their normal counterparts.

### PinX1 suppresses cell proliferation in 12 types of cancer cell lines *in vitro*

**Figure 6:**
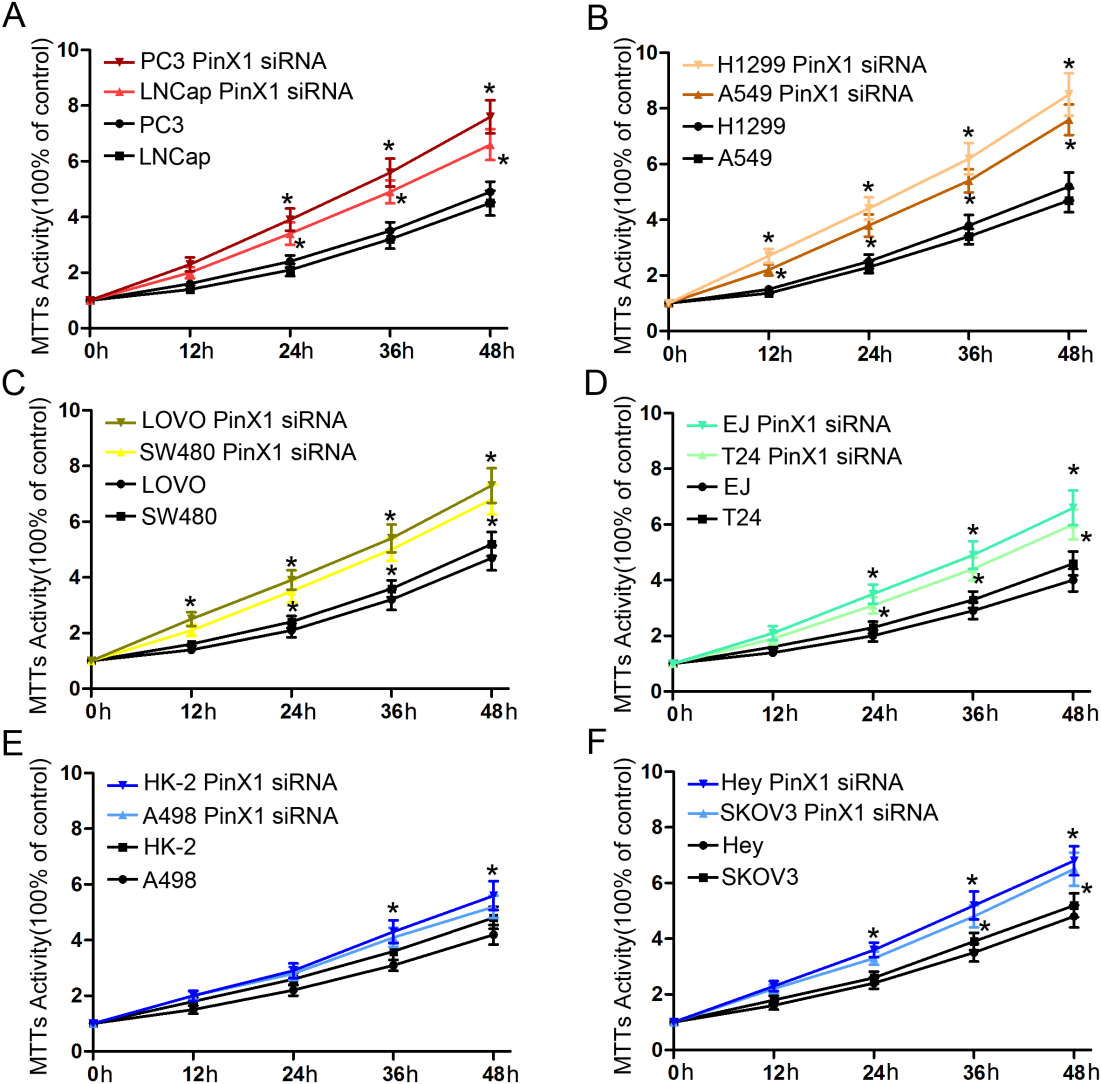
*PinX1* suppresses cell proliferation of 12 types of cancer cell lines *in vitro* MTT analysis was used to detect cell proliferation in 12 types of cancer transfected with PinX1 siRNA. **(A)** prostate cancer cells (PC3 and LNCap cell lines); **(B)** lung cancer cells (H1299 and A549 cell lines); **(C)** colorectal cancer cells (LOVO and SW480 cell lines); **(D)** bladder cancer cell (EJ and T24 cell lines); **(E)** kidney cancer cell (HK-2 and A498 cell lines); **(F)** ovarian cancer cell (Hey and SKOV3 cell lines). *, *P*<0.05.

We then continued our study *in vitro* to investigate whether knock-down of PinX1 expression promotes cell proliferation in cancer cell lines. In our MTT analysis, prostate cancer cells (PC3 and LNCap cell lines) (Figure 6A), lung cancer cells (H1299 and A549 cell lines) (Figure 6B), colorectal cancer cells (LOVO and SW480 cell lines) (Figure 6C), bladder cancer cells (EJ and T24 cell lines) (Figure 6D), and ovarian cancer cells (Hey cell line) (Figure 6F) were all separately transfected with PinX1 siRNA. After 24 h, all cell lines displayed a significant increase in cell proliferation when compared with control cells (*P*<0.05). However, cell proliferation in both kidney (HK-2 cell line) (Figure 6E) and ovarian (SKOV3 cell line) (Figure 6F) cancer cells transfected with PinX1 siRNA had significantly increased proliferation until 36 h after transfection (*P*<0.05). There were no obvious differences in cell proliferation between kidney cancer cells (A498 cell line) (Figure 6E) and their corresponding controls (*P*>0.05).

## DISCUSSION

Previous work has shown that the telomerase-inhibitory domain of PinX1-C (amino acids 254-328) can block telomerase activity, shorten telomeres, induce cellular dysfunction, and suppress tumor growth [16]. However, PinX1 protein expression differs greatly depending on the type of tumor tissue. For example, one study demonstrated that high expression of PinX1 in esophageal squamous cell carcinoma is associated with a patient’s chemoradiotherapy resistance and can predict his/her survival [15]. However, another study showed that PinX1 expression in bladder urothelial carcinoma was significantly down-regulated at both the mRNA and protein levels when compared with normal tissue [17]. Given these apparently conflicting findings, it is fair to say the function of PinX1 is complicated. It has been previously reported as a potent telomerase inhibitor. However, recent reports suggest that PinX1 can also function in chemotherapy and radiotherapy resistance, act as a co-regulator of nuclear hormone receptors, participate in cell cycle regulation, and engage in chromosome segregation [18].

The cBioportal for Cancer Genomics is an open-access resource for interactive exploration of multidimensional cancer genomics data sets. It currently provides access to data from more than 5,000 tumor samples from 20 cancer studies. Importantly, the cBio Cancer Genomics Portal allows cancer researchers the ability to use complex genomic data, thereby ensuring rapid, intuitive, and high-quality access to molecular profiles and clinical attributes from large-scale cancer genomics projects. Ultimately, this empowers researchers to translate rich data sets into biological insights and clinical applications [19, 20].

In this study, we explored PinX1 gene alterations in 105 studies using the online resource cBioportal Web. Our results indicated that PinX1 deletion is a common genotype in human cancer patients, leading us to believe that insufficient PinX1 may be involved in the progression of a variety of human cancers. In addition, we also observed that the genotype of PinX1 gene deletion often occurred in both carcinosarcoma and adenocarcinoma. These results implied that the abnormalities and/or functions of PinX1 in tumorigenesis and its progression may be pathologically specific. With the exception of the high frequency of *PinX1* gene deletion, we also found that *PinX1* genetic mutations occurred in some types of human cancers, including bladder urothelial carcinoma, lung adenocarcinoma, and melanoma. However, the number of cases having a PinX1 gene mutation was very few. It is worth noting that these mutation sites were mostly concentrated within the amino acids 150 and 328. This region mainly contains both the nucleolar localization and telomerase inhibitor domains, which are associated with TERT. More importantly, these regions can specifically bind Pin2/TRF1 (PinX1) [21]. Additional work performing 3D structural analysis showed that that these mutation sites may influence this structural domain, which is specifically recognized by the TRFH domain of TRF1 via both hydrophobic and hydrophilic interactions [22]. Thus, such mutations could break the PinX1-TRF1-TERT complex, thereby promoting telomere elongation in cells. These results imply that PinX1 function in tumorigenesis and tumor progression might be all or partly related to loss of telomerase inhibition. This would ultimately promote telomere elongation. However, it is still not clearly defined that PinX1 tumorigenicity occurs via telomere-dependent and/or telomere-independent telomerase. It is possible that other mechanisms are involved that are unrelated to the actions of telomerase.

Since the deletion of *PinX1* accounts for the most observed alterations, we next randomly selected six studies to better understand the relationship between *PinX1* gene copy number and mRNA levels. Among the five types of copy numbers we examined (deep deletion, shallow deletion, diploid, gain and amplification), we found that the frequency of *PinX1* homozygous depletion as well as *PinX1* heterozygous deficiency were the main reasons for the frequency of *PinX1* deletion in a wide variety of tumor tissues. These results showed that *PinX1* has a frequent loss of heterozygosity (LOH) at 8p23 in human malignancies [16, 22]. The remaining copy number contains insufficient PinX1 for full inhibition of telomerase activity, which could immortalize the tumor and result in telomere lengthening. According to our mutation data, *PinX1* mutations in gastric, colorectal, prostate, breast, and lung carcinomas may not actually contribute to development of these carcinomas due to their low incidence of mutational events seen in the database. When taken together, these results suggest that somatic mutation is not the mechanism for PinX1 inactivation [23]. Rather, loss of hetereozygosity seems to play a major role in the inactivation of PinX1 in human cancers.

Since PinX1 is a putative tumor suppressor, we next sought to determine whether it could serve as an indicator for patient survival. Thus, we used a Kaplan-Meier analysis to determine the relationship between PinX1 gene alterations and patient survival. Our results demonstrated that PinX1 gene alterations were correlated with poor survival in patients with lung adenocarcinoma (overall survival and disease-free survival) and as well as those diagnosed with bladder urothelial carcinoma (disease-free survival only). However, there were no significant correlations between *PinX1* alterations and overall survival and/or disease-free survival in the other seven types of tested human cancers. These results showed that the status of *PinX1* gene alteration was correlated with prognosis; importantly, that this correlation may be tumor-type specific. Furthermore, this indicates that its biological function in tumorigenesis and prognosis is complicated and mainly co-regulated with other factors.

Given this likely co-regulatory influence, we then examined the relationship between other genes and *PinX1*. Using the cBioportal database, we found that NEIL2, R3HCC1, POLR3D, GTF2E2, and INTS10 were the most common genes that had positive correlations with *PinX1* between the seven selective studies. NEIL2 initiates the first step in base excision repair by cleaving bases damaged by reactive oxygen species. It does so by introducing a DNA strand break via an associated lyase reaction [24]. POLR3D leads to a block in progression through the G1 phase of the cell cycle [25]. GTF2E2 is also named general transcription factor IIE (TFIIE), which is part of the RNA polymerase II transcription initiation complex. It recruits TFIIH and is essential for promoter clearance by RNA polymerase II [26]. INTS10 is a subunit of the Integrator complex, which associates with the C-terminal domain of RNA polymerase II large subunit and mediates 3-prime end processing of small nuclear RNAs U1 and U2 [27]. In addition, we also explored the PinX1 integrated network using the cBioportal. We found that PinX1 interacts with TERT, DCK1, PTGES3, and HSP90AA1, which are mainly connected with telomerase maintenance, DNA damage, and steroid receptors [28–31]. However, we have only determined their place in a wider network; their precise mechanism of action will need further research.

Finally, we selected nine types of cancer samples from our affiliated hospitals to verify their respective PinX1 mRNA expression status. When compared with normal tissue, PinX1 mRNA expression was significantly decreased in most of the selected cancer tissues. It has been reported that PinX1 suppress tumor growth and depletion of endogenous PinX1 can enhance tumorigenicity [32, 33]. We also observed that knockdown of PinX1 could also enhance cell proliferation in nine types of cancer cell lines *in vitro*.

Although our data provide additional information regarding PinX1, its exact mechanism and function in human cancer cells has yet to be fully elucidated. PinX1 was originally identified as a telomerase inhibitor and is involved in maintaining telomerase activity, telomere length, and chromosomal stability. However, PinX1 has also been shown to interact during mitosis with the outer plate of kinetochores and the periphery of chromosomes [34, 35]. Thus, loss of PinX1 abrogates faithful chromosome segregation, suggesting a novel function outside of its established roles [12]. Furthermore, the regulation of telomerase activity by PinX1 is also involved in the Mad/c-Myc and NF-κB pathways along with the cell-cycle [36–38]. Given this, further work will be needed to clarify the mechanisms of PinX1 in regulation of tumorigenesis and the progression of different types of human carcinomas.

## MATERIALS AND METHODS

### Analysis of cancer genomics using the open-access bio-database cBioportal

The cBioPortal for Cancer Genomics provided visualization, analysis, and the ability to download large-scale cancer genomics data sets (http://cbioportal.org). This portal collects records that were derived from 150 individual cancer studies, in which 31 types of cancer were analyzed. Collectively, the data sets include over 21,000 samples [39]. The term “PinX1” was searched in the cBioportal for Cancer Genomics database and a cross-cancer summary was obtained for it. Analysis of PinX1 gene alterations, mutation levels, genetic profiles, 3D structural analyses, clinical impacts, gene co-expression, and integrated networks from this database was then performed *in silico*.

### Patients and tissue specimens

Nine types of cancer tissue (Prostate Adenocarcinoma, Colorectal Adenocarcinoma, Head and Neck Squamous Cell Carcinoma, Kidney Renal Clear Cell Carcinoma, Lung Adenocarcinoma, Lung Squamous Cell Carcinoma, Bladder Urothelial Carcinoma, Ovarian Serous Cystadenocarcinoma, Esophagus Carcinoma) from 108 patients were provided by the Department of Pathology, Cancer Center, Sun Yat-Sen University between January 2015 and January 2016. All cancer specimens were independently reviewed and evaluated for histological type by LM and JXH. All tissue specimens were harvested in a similar manner: Specimens were surgically extracted, immediately frozen with liquid nitrogen, and stored at −80°C until later analysis. All patients provided written, informed consent and all aspects of the study were approved by the Medical Ethics Committee of the Department of Pathology.

### Quantitative real-time polymerase chain reaction (qRT-PCR) analysis

The construction of PinX1 and GAPDH sense/antisense primers has been previously described. Briefly, RNA was reverse-transcribed using SuperScript First Strand cDNA System (Invitrogen, USA) according to the manufacturer’s instructions. qRT-PCR was performed using Real-time PCR system (Applied Biosystems, USA) according to the following thermocycling protocol: 50°C for 2 min, 95°C for 10 min, 40 cycles of 95°C for 15 s, and 60°C for 60 s. The relative levels of gene expression were represented as ΔCt = Ct_gene_- Ct_reference_ and the fold change for gene expression was calculated using the 2–ΔΔCt Method.

### Cell lines and PinX1 siRNA sequence

PC3, LNCap, H1299, A549, LOVO, SW480, EJ, T24, HK-2, A498, Hey, and SKOV3 cells were maintained in DMEM and/or RPMI 1640 supplemented with 10% fetal bovine serum and 1% penicillin–streptomycin at 37°C in 5% CO_2_. The PinX1 siRNA transient transfection (GGAGCTACCATCAATAATG) was used to transiently decrease PinX1 expression.

### MTT proliferation assay

Cellular viability was measured using the MTT proliferation assay according to the manufacturer’s instructions. Briefly, 1000 cells were seeded in 96-well plates and cultured/treated for 24 h. Viability was measured at different time points post-treatment, ranging from 12 h to 48 h and based on the experimental requirements.

### Statistical analysis

Survival curves were plotted using a Kaplan-Meier analysis and compared using the log-rank test. The correlation between expression of PinX1 and co-expression of other gene was performed using both Pearson’s and Spearman’s correlations. Data derived from cell line experiments are presented as mean ± SE and were assessed using a Student’s t test. A *P* value of < 0.05 was considered to be statistically significant.

## SUPPLEMENTARY MATERIALS FIGURE AND TABLE





## References

[1] Martinez P, Blasco MA (2017). Telomere-driven diseases and telomere-targeting therapies. J Cell Biol.

[2] Qin Y, Guo H, Tang B, Yang SM (2014). The non-reverse transcriptase activity of the human telomerase reverse transcriptase promotes tumor progression. Int J Oncol.

[3] Bellon M, Nicot C (2008). Regulation of telomerase and telomeres: human tumor viruses take control. J Natl Cancer Inst.

[4] Belgiovine C, Chiodi I, Mondello C (2008). Telomerase: cellular immortalization and neoplastic transformation. Multiple functions of a multifaceted complex. Cytogenet Genome Res.

[5] Chen CH, Chen RJ (2011). Prevalence of telomerase activity in human cancer. J Formos Med Assoc.

[6] Bandaria JN, Qin P, Berk V, Chu S, Yildiz A (2016). Shelterin protects chromosome ends by compacting telomeric chromatin. Cell.

[7] Schmidt JC, Dalby AB, Cech TR (2014). Identification of human TERT elements necessary for telomerase recruitment to telomeres. Elife.

[8] Zhou XZ, Lu KP (2001). The Pin2/TRF1-interacting protein PinX1 is a potent telomerase inhibitor. Cell.

[9] Sun C, Wu Z, Jia F, Wang Y, Li T, Zhao M (2008). Identification of zebrafish LPTS: a gene with similarities to human LPTS/PinX1 that inhibits telomerase activity. Gene.

[10] Kishi S, Lu KP (2002). A critical role for Pin2/TRF1 in ATM-dependent regulation. Inhibition of Pin2/TRF1 function complements telomere shortening, radiosensitivity, and the G(2)/M checkpoint defect of ataxia-telangiectasia cells. J Biol Chem.

[11] Lin J, Blackburn EH (2004). Nucleolar protein PinX1p regulates telomerase by sequestering its protein catalytic subunit in an inactive complex lacking telomerase RNA. Genes Dev.

[12] Yuan K, Li N, Jiang K, Zhu T, Huo Y, Wang C, Lu J, Shaw A, Thomas K, Zhang J, Mann D, Liao J, Jin C, Yao X (2009). PinX1 is a novel microtubule-binding protein essential for accurate chromosome segregation. J Biol Chem.

[13] Zhang B, Bai YX, Ma HH, Feng F, Jin R, Wang ZL, Lin J, Sun SP, Yang P, Wang XX, Huang PT, Huang CF, Peng Y (2009). Silencing PinX1 compromises telomere length maintenance as well as tumorigenicity in telomerase-positive human cancer cells. Cancer Res.

[14] Cai MY, Zhang B, He WP, Yang GF, Rao HL, Rao ZY, Wu QL, Guan XY, Kung HF, Zeng YX, Xie D (2010). Decreased expression of PinX1 protein is correlated with tumor development and is a new independent poor prognostic factor in ovarian carcinoma. Cancer Sci.

[15] Qian D, Zhang B, He LR, Cai MY, Mai SJ, Liao YJ, Liu YH, Lin MC, Bian XW, Zeng YX, Huang JJ, Kung HF, Xie D (2013). The telomere/telomerase binding factor PinX1 is a new target to improve the radiotherapy effect of oesophageal squamous cell carcinomas. J Pathol.

[16] Johnson FB (2011). PinX1 the tail on the chromosome. J Clin Invest.

[17] Liu JY, Qian D, He LR, Li YH, Liao YJ, Mai SJ, Tian XP, Liu YH, Zhang JX, Kung HF, Zeng YX, Zhou FJ, Xie D (2013). PinX1 suppresses bladder urothelial carcinoma cell proliferation via the inhibition of telomerase activity and p16/cyclin D1 pathway. Mol Cancer.

[18] Li HL, Song J, Yong HM, Hou PF, Chen YS, Song WB, Bai J, Zheng JN (2016). PinX1: structure, regulation and its functions in cancer. Oncotarget.

[19] Gao J, Aksoy BA, Dogrusoz U, Dresdner G, Gross B, Sumer SO, Sun Y, Jacobsen A, Sinha R, Larsson E, Cerami E, Sander C, Schultz N (2013). Integrative analysis of complex cancer genomics and clinical profiles using the cBioPortal. Sci Signal.

[20] Noriega-Reyes MY, Rivas-Torres MA, Onate-Ocana LF, Valles AJ, Baranda-Avila N, Langley E (2015). Novel role for PINX1 as a coregulator of nuclear hormone receptors. Mol Cell Endocrinol.

[21] Cheung DH, Ho ST, Lau KF, Jin R, Wang YN, Kung HF, Huang JJ, Shaw PC (2017). Nucleophosmin interacts with PIN2/TERF1-interacting telomerase inhibitor 1 (PinX1) and attenuates the PinX1 inhibition on telomerase activity. Sci Rep.

[22] Zhou XZ (2011). PinX1: a sought-after major tumor suppressor at human chromosome 8p23. Oncotarget.

[23] Kim MS, Kim SS, Yoo NJ, Lee SH (2012). Somatic mutation of PINX1 gene is rare in common solid cancers. APMIS.

[24] Dou H, Mitra S, Hazra TK (2003). Repair of oxidized bases in DNA bubble structures by human DNA glycosylases NEIL1 and NEIL2. J Biol Chem.

[25] Ittmann MM (1994). Cell cycle control of the BN51 cell cycle gene which encodes a subunit of RNA polymerase III. Cell Growth Differ.

[26] Kuschal C, Botta E, Orioli D, Digiovanna JJ, Seneca S, Keymolen K, Tamura D, Heller E, Khan SG, Caligiuri G, Lanzafame M, Nardo T, Ricotti R (2016). GTF2E2 mutations destabilize the general transcription factor complex TFIIE in individuals with DNA repair-proficient trichothiodystrophy. Am J Hum Gene.

[27] Baillat D, Hakimi MA, Naar AM, Shilatifard A, Cooch N, Shiekhattar R (2005). Integrator, a multiprotein mediator of small nuclear RNA processing, associates with the C-terminal repeat of RNA polymerase II. Cell.

[28] Yoo JE, Park YN, Oh BK (2014). PinX1, a telomere repeat-binding factor 1 (TRF1)-interacting protein, maintains telomere integrity by modulating TRF1 homeostasis, the process in which human telomerase reverse Transcriptase (hTERT) plays dual roles. J Biol Chem.

[29] Hope H, Bogliolo S, Arkowitz RA, Bassilana M (2008). Activation of Rac1 by the guanine nucleotide exchange factor Dck1 is required for invasive filamentous growth in the pathogen Candida albicans. Mol Biol Cell.

[30] Patel N, Crider A, Pandya CD, Ahmed AO, Pillai A (2016). Altered mRNA levels of glucocorticoid receptor, mineralocorticoid receptor, and co-chaperones (FKBP5 and PTGES3) in the middle frontal gyrus of autism spectrum disorder subjects. Mol Neurobiol.

[31] Marcos-Carcavilla A, Calvo JH, Gonzalez C, Moazami-Goudarzi K, Laurent P, Bertaud M, Hayes H, Beattie AE, Serrano C, Lyahyai J, Martin-Burriel I, Serrano M (2008). Structural and functional analysis of the HSP90AA1 gene: distribution of polymorphisms among sheep with different responses to scrapie. Cell Stress Chaperones.

[32] Mei PJ, Chen YS, Du Y, Bai J, Zheng JN (2015). PinX1 inhibits cell proliferation, migration and invasion in glioma cells. Med Oncol.

[33] Zhou XZ, Huang P, Shi R, Lee TH, Lu G, Zhang Z, Bronson R, Lu KP (2011). The telomerase inhibitor PinX1 is a major haploinsufficient tumor suppressor essential for chromosome stability in mice. J Clin Invest.

[34] Cheung DH, Kung HF, Huang JJ, Shaw PC (2012). PinX1 is involved in telomerase recruitment and regulates telomerase function by mediating its localization. FEBS Lett.

[35] Tian XP, Qian D, He LR, Huang H, Mai SJ, Li CP, Huang XX, Cai MY, Liao YJ, Kung HF, Zeng YX, Xie D (2014). The telomere/telomerase binding factor PinX1 regulates paclitaxel sensitivity depending on spindle assembly checkpoint in human cervical squamous cell carcinomas. Cancer Lett.

[36] Wang HB, Wang XW, Zhou G, Wang WQ, Sun YG, Yang SM, Fang DC (2010). PinX1 inhibits telomerase activity in gastric cancer cells through Mad1/c-Myc pathway. J Gastrointest Surg.

[37] Li HL, Han L, Chen HR, Meng F, Liu QH, Pan ZQ, Bai J, Zheng JN (2015). PinX1 serves as a potential prognostic indicator for clear cell renal cell carcinoma and inhibits its invasion and metastasis by suppressing MMP-2 via NF-kappaB-dependent transcription. Oncotarget.

[38] Shi M, Cao M, Song J, Liu Q, Li H, Meng F, Pan Z, Bai J, Zheng J (2015). PinX1 inhibits the invasion and metastasis of human breast cancer via suppressing NF-kappaB/MMP-9 signaling pathway. Mol Cancer.

[39] Cerami E, Gao J, Dogrusoz U, Gross BE, Sumer SO, Aksoy BA, Jacobsen A, Byrne CJ, Heuer ML, Larsson E, Antipin Y, Reva B, Goldberg AP (2012). The cBio cancer genomics portal: an open platform for exploring multidimensional cancer genomics data. Cancer Discov.

