# Supplementary material for: Genetic profile and biological implication of PIN2/TRF1-interacting telomerase inhibitor 1 (PinX1) in human cancers: an analysis using The Cancer Genome Atlas
Source: Oncotarget. 2017 Jun 21;8(40):67241–53. doi: 10.18632/oncotarget.18589 (PMC5620170; doi:10.18632/oncotarget.18589)
Supplement: Supplementary file 1 [file oncotarget-08-67241-s001.pdf]

Genetic profile and biological implication of PIN2/TRF1-interacting telomerase inhibitor 1 (PinX1) in human cancers: an analysis using The Cancer Genome Atlas

SUPPLEMENTARY MATERIALS

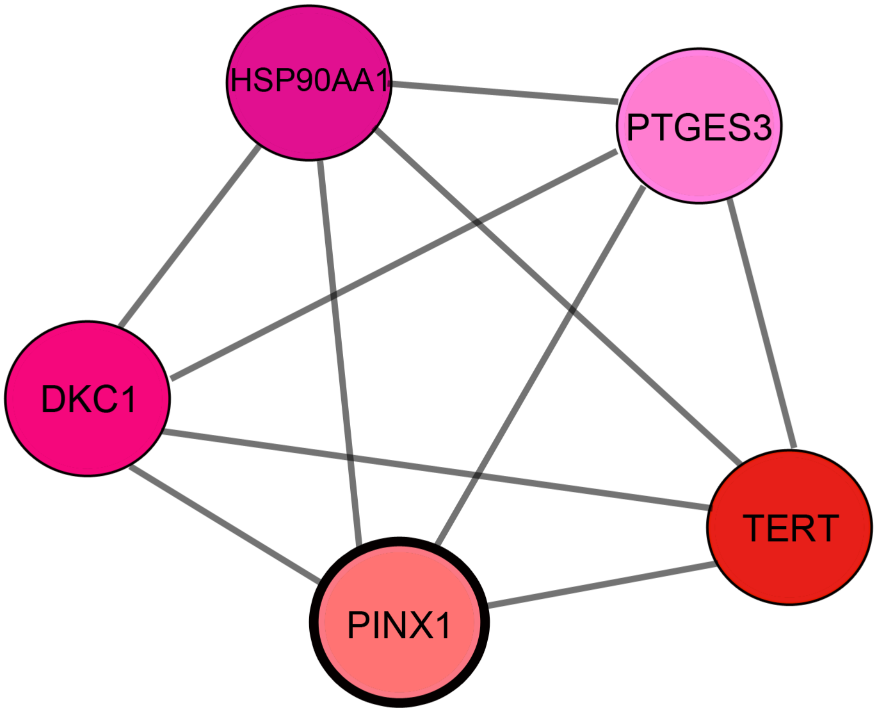

Supplementary Figure 1: Integrated network to evaluate PinX1 gene connectivity using cBioportal for Cancer genomics. The cBioportal for Cancer genomics network was used to evaluate PinX1 gene connectivity. PinX1 interacts with TERT, DKC1, PTGES3 and HSP90AA1 from the parallel distributed algorithms. TERT is connected to telomerase activity and telomerase stability. DKC1 is connected to telomerase maintenance, DNA damage response, and cell adhesion. PTGES3 is required for proper functioning of glucocorticoid and other steroid receptors. HSP90AA1 primarily participates in stabilizing a number of proteins required for tumor growth.

Supplementary Table 1: Ten of the most closely related (positively or negatively correlated) genes with *PinX1* are explored in lung adenocarcinoma and lung squamous cell carcinoma

	Positive correlation with *PinX1* gene	Negative correlation with *PinX1* gene
Lung Adenocarcinoma (TCGA, Provisional)	MRGPRD, PSMA3, TTI2, TIMM9, SEC61B, MAK16, NTMT1, ERH, HSPE1, AKR1C3, et al.	HIPK1, ATXN1, AAK1
Lung Squamous Cell Carcinoma (TCGA, Provisional)	CCDC25, PPP2R2A, BIN3, ESCO2, XPO7, CCAR2, CDCA2, INTS9, RPL23AP53, TNFRSF10A, et al.	none

Supplementary Data 1: PinX1 genetic mutation in 105 studies using cBioportal Web

See Supplementary File 1
